# Regulation of Muscle Stem Cell Functions: A Focus on the p38 MAPK Signaling Pathway

**DOI:** 10.3389/fcell.2016.00091

**Published:** 2016-08-30

**Authors:** Jessica Segalés, Eusebio Perdiguero, Pura Muñoz-Cánoves

**Affiliations:** ^1^Cell Biology Group, Department of Experimental and Health Sciences, CIBER on Neurodegenerative diseases (CIBERNED), Pompeu Fabra UniversityBarcelona, Spain; ^2^Institució Catalana de Recerca i Estudis Avançats (ICREA)Barcelona, Spain; ^3^Tissue Regeneration Laboratory, Centro Nacional de Investigaciones CardiovascularesMadrid, Spain

**Keywords:** muscle stem cells, satellite cells, p38 MAPKs, aging, epigenetics, tissue regeneration

## Abstract

Formation of skeletal muscle fibers (myogenesis) during development and after tissue injury in the adult constitutes an excellent paradigm to investigate the mechanisms whereby environmental cues control gene expression programs in muscle stem cells (satellite cells) by acting on transcriptional and epigenetic effectors. Here we will review the molecular mechanisms implicated in the transition of satellite cells throughout the distinct myogenic stages (i.e., activation from quiescence, proliferation, differentiation, and self-renewal). We will also discuss recent findings on the causes underlying satellite cell functional decline with aging. In particular, our review will focus on the epigenetic changes underlying fate decisions and on how the p38 MAPK signaling pathway integrates the environmental signals at the chromatin to build up satellite cell adaptive responses during the process of muscle regeneration, and how these responses are altered in aging. A better comprehension of the signaling pathways connecting external and intrinsic factors will illuminate the path for improving muscle regeneration in the aged.

## Introduction

The regenerative capacity of adult skeletal muscle depends on a resident population of muscle stem cells called satellite cells, which are located between the sarcolemma and the basal lamina. In healthy adult muscles, satellite cells reside in a dormant quiescent state and are characterized by the expression of the paired-box transcription factor Pax7 (Chang and Rudnicki, 2014; Comai and Tajbakhsh, 2014). Different stimuli, such as injury or disease, are known to induce satellite cell activation from quiescence and expansion as myoblasts; these cells will subsequently either exit the cell cycle, differentiate and fuse to generate new muscle fibers (i.e., myogenesis) or undergo self-renewal and return to quiescence to replenish the stem cell pool (Brack and Rando, 2012; Wang et al., 2014).

The transcriptional regulation of muscle cell specification has been well characterized. Pax3 and Pax7 are thought to be the principal regulators of muscle cell specification and tissue formation during embryonic development. In adult satellite cells, whereas Pax7 is highly expressed, Pax3 is only highly expressed in satellite cells of a subset of muscles, such as the diaphragm, but lowly expressed in other muscles (Soleimani et al., 2012a). Recent studies analyzing the role of Pax7 in the maintenance and expansion of adult muscle stem cells have demonstrated that long-term Pax7 ablation leads to the loss of adult satellite cells, which results in impaired muscle regeneration after injury confirming that Pax7^+^ satellite cells are sufficient and required for adult muscle repair (Lepper et al., 2011; Sambasivan et al., 2011; von Maltzahn et al., 2013; Brack, 2014). Furthermore, inactivation of Pax7 in satellite cells of adult mice also leads to diminished heterochromatin condensation (Gunther et al., 2013), suggesting a role for Pax7 in chromatin organization; of note, this has also been recently proposed for Pax3 (Bulut-Karslioglu et al., 2012).

Satellite cells can be activated by many signals from the regenerative microenvironment, including those mediated by adhesion molecules, necrotic cues released from the damaged fibers, or by growth factors and cytokines produced by neighboring cells, including interstitial cells, resident macrophages, fibroblasts, and microvasculature-related cells (Giordani and Puri, 2013; Judson et al., 2013; Pannerec et al., 2013; Brancaccio and Palacios, 2015). Several signaling cascades, including the p38 mitogen-activated protein kinase (MAPK) and the AKT pathways, can transmit these extracellular cues to the myogenic cell nucleus (Cuenda and Cohen, 1999; Wu et al., 2000; Keren et al., 2006; Serra et al., 2007), and regulate expression and activity of the muscle-specific regulatory factors (MRFs, that belong to the bHLH family of transcription factors: Myf5, MyoD, myogenin and MRF4). In cooperation with ubiquitously-expressed E proteins and myocyte enhancer factor 2 (MEF2) transcriptional regulators, MRFs bind to E-boxes and MEF2-boxes of muscle promoters and induce the expression of muscle-specific genes (Lluis et al., 2006; Singh and Dilworth, 2013; Segales et al., 2015). Satellite cell-dependent myogenesis is also controlled by several epigenetic mechanisms, such as chromatin remodeling, DNA methylation and covalent modification of histones and transcription factors. The cross-talk between the basic muscle-specific transcriptional and epigenetic machineries allows the coordinated induction or repression of distinct subsets of genes in order to advance through the myogenic program (Dilworth and Blais, 2011; Segales et al., 2015).

In this review, we will sum up the existing knowledge about the transcriptional and epigenetic regulation of postnatal myogenesis, describing the epigenetic status of adult muscle stem cells and the changes in chromatin structure needed to coordinately regulate the myogenic gene expression program. We will also discuss the main signal transduction pathways that transmit extracellular signals to the nucleus of myogenic cells, focusing especially on the p38 MAPK pathway. Finally, we will address how alterations in these signaling cascades contribute to the reduced regenerative capacity of aged satellite cells and whether this defect can be counteracted by pharmacological manipulation of these pathways.

## Transcriptional and translational regulation of myogenesis

Myogenesis dependent on satellite cells is a well-defined multi-step process characterized by the sequential activation of MRFs: Myf5 and MyoD are expressed in undifferentiated proliferating myoblasts, while myogenin and MRF4 are induced at the early and late phases of differentiation, respectively (Sartorelli and Caretti, 2005; Singh and Dilworth, 2013). Pax7 is expressed in quiescent satellite cells and is also critical for their cell cycle progression by regulating genes involved in cell proliferation, while preventing differentiation (Olguin et al., 2007; Soleimani et al., 2012a; von Maltzahn et al., 2013). Pax7-expressing quiescent satellite cells induce the expression of Myf5 and MyoD upon their activation (Mckinnell et al., 2008), thereby allowing successive rounds of cell proliferation (Olguin and Olwin, 2004); instead, downregulation of Pax7 prior to myogenin activation facilitates exit the cell cycle and differentiation entry (Olguin et al., 2007; Olguin, 2011; Bustos et al., 2015). Translational control of MRFs' expression also accounts for the transition through the sequential myogenic stages: (1) In quiescent satellite cells the expression of the Myf5 protein is avoided by sequestration of the Myf5 mRNA in messenger ribonucleoprotein granules and by the action of the microRNA-31, which blocks Myf5 translation (Crist et al., 2012); (2) MyoD protein expression is also prevented in quiescent satellite cells by the action of tristetraprolin (TTP), a protein that promotes the degradation of MyoD RNA (Hausburg et al., 2015); (3) Moreover, a global mechanism of repression of translation, mediated by the phosphorylation of the eukaryotic initiation factor eIF2α at serine 51, preserves the quiescent state of satellite cells, as cells that cannot phosphorylate eIF2α exit quiescence and activate the myogenic program (Zismanov et al., 2016).

Once MyoD mRNA has been correctly translated, the initiation of the differentiation program requires the association of MyoD with E proteins and their binding to E boxes of muscle gene promoters, to activate their expression. This is actively inhibited in proliferating myoblasts by different mechanisms. Initially, in proliferating myoblasts, the formation of functional MyoD/E protein heterodimers is prevented by the inhibitor of differentiation (Id), an HLH protein that lacks the basic DNA-binding domain and interacts with either MyoD or E proteins, thus inhibiting myogenic differentiation. When myoblasts exit the cell cycle Id expression is downregulated, allowing functional heterodimers to be formed and promoting muscle differentiation-specific gene expression (Puri and Sartorelli, 2000). Other proteins, such as the bHLH factors Twist, masculine, Mist1, ZEB, Hey1, and I-mfa proteins, act as repressors of MRFs either by direct association with them or by sequestering their functional partners (Buas et al., 2010; Siles et al., 2013). Moreover, in proliferating myoblasts, the transcriptional repressor Snai1 excludes MyoD from muscle-specific promoters. During differentiation, Snai1/2 expression is downregulated by the action of two specific microRNAs, miR-30 and miR-206, allowing access for MyoD to its target genes (Soleimani et al., 2012b).

Interestingly, recent genome-wide analyses of MyoD binding in C2C12 myogenic cells and primary myoblasts have revealed that MyoD also binds to a large number of promoters of genes that are not regulated during muscle differentiation, in both myoblasts and myotubes (Cao et al., 2010; Soleimani et al., 2012b). This genome-wide MyoD binding correlates with local histone hyperacetylation, suggesting that MyoD could play a role in reprogramming the epigenetic landscape by recruiting histone acetyltransferases (HATs) to regions throughout the genome.

## Epigenetic regulation of myogenesis

As mentioned above, muscle gene expression is also regulated by different epigenetic mechanisms, including DNA methylation, chromatin remodeling, post-translational modifications of histones and regulation by a network of noncoding RNA. These epigenetic modifications are usually dynamic and reversible and can be associated with gene activation or repression (reviewed in Bergman and Cedar, 2013).

## Epigenetic status of quiescent satellite cells

**Figure 1:**
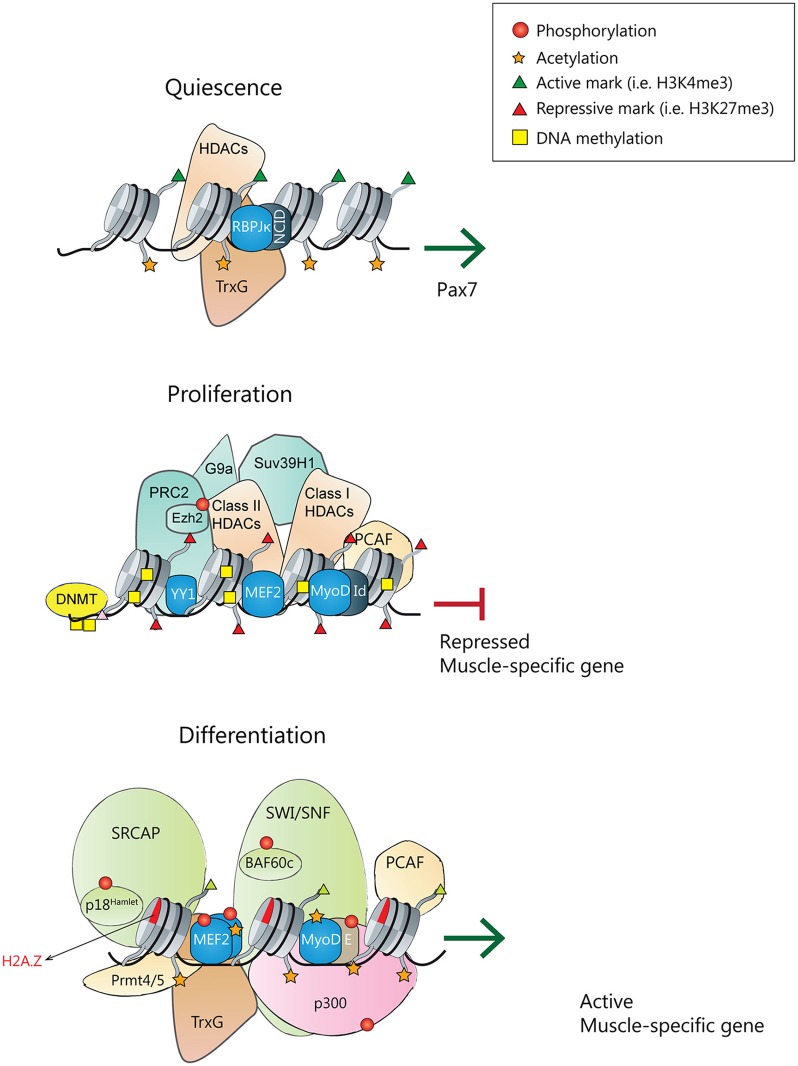
**Transcriptional and epigenetic regulators of satellite cell quiescence, proliferation and differentiation. (Top)** During homeostasis, quiescent satellite cells express Pax7. Pax7 promoter is active, holding active chromatin marks, and being transcriptionally regulated by the Notch signaling pathway with the Notch intracellular domain (NICD) interacting with the effector protein recombining binding protein-Jκ (RBPJκ) (Wen et al., 2012), and although not demonstrated, probably populated by active chromatin remodelers and HATs. **(Middle)** In quiescent and proliferating satellite cells, muscle-specific gene promoters are repressed. MyoD is associated with several repressors (like Id) and Sir2 in a complex that also contains pCAF. MyoD, YY1, and MEF2 factors recruit the PRC2 complex, Suv39H1, and class I/II HDACs. DNMTs associate and methylate the DNA, and chromatin is populated with repressive histone marks. **(Bottom)** Upon differentiation cues, transcriptionally active muscle-specific promoters contain active phosphorylated MyoD/E heterodimers, phosphorylated MEF2 dimers and SRF transcription factors. In collaboration with arginine methyltransferases Prmt4/5, the SWI/SNF remodeling complex, HATs and Thritorax complexes will be recruited. DNA will be demethylated, and chromatin acetylated and populated with active histone marks.

The capacity to maintain quiescence is essential for the long-term preservation of a functional stem cell pool in the majority of tissues and organs. In stem cells, genes implicated in lineage determination and progression are found in a poised state: although transcriptionally silent, they contain active histone marks that keep them prepared to be expressed after receiving differentiation signals (Puri et al., 2015; Rumman et al., 2015). In quiescent satellite cells, chromatin is kept in a transcriptionally permissive state, with many genes harboring the activation H3K4me3 mark at the transcription start sites (TSS), including MyoD and Myf5, and a few number of genes labeled with the inhibitory H3K27me3 mark (Liu et al., 2013). Thus, instead of being only found on actively transcribed genes, the H3K4me3 mark is also present in inactive genes that will likely be transcribed at later time points, for example, following satellite cell activation (Guenther et al., 2007). Furthermore, Liu and colleagues have also described that quiescent satellite cells contain more than 1800 genes with their transcription start site (TSS) marked by bivalent domains (e.g., both H3K27me3 and H3K4me3 marks), many of them corresponding to lineage-specific genes (Mikkelsen et al., 2007). Of interest, in quiescent satellite cells, the only myogenic transcription factor having bivalent domains is *Pax3* whereas, *Pax7* is only marked by H3K4me3 (Figure 1). In contrast, myogenin which is not marked by either H3K4me3 or H3K27me3 in quiescent satellite cells, shows a significant enrichment of the H3K4me3 mark at its TSS upon cell activation (Liu et al., 2013). Together, these data suggest an interplay between the Trithorax complex (TrxG; responsible of H3K4me3) and the polycomb repressive complexes (PRCs; responsible of H3K27me3). Additionally, H3K9 methyltransferase PRDM2/RIZ, which is highly expressed in quiescent satellite cells, binds to thousands of promoters in G0 synchronized C2C12 myoblasts, including myogenic and cell cycle regulators (Cheedipudi et al., 2015a,b). PRDM2 interacts with Ezh2, the catalytic subunit of PRC2, and regulates its association with a novel G0-specific bivalent domain identified in the Ccna2 locus (Cheedipudi et al., 2015a). Ezh2, in turn, is needed for homeostasis of the adult muscle stem cell pool (Juan et al., 2011). Mice lacking Ezh2 specifically in satellite cell have reduced muscle mass, fewer satellite cells post-birth, and impaired regeneration following muscle injury. These differences can be explained by defects in the proliferative capacity of satellite cells (Woodhouse et al., 2013), and by impaired maintenance and/or return to quiescence after injury (Juan et al., 2011). Moreover, recent studies showed that preservation of muscle stem cell quiescence is also dependent on the repression of senescence pathways by Polycomb proteins (Sousa-Victor et al., 2014a). Indeed, derepression of the senescence regulator p16^INK4a^ (*Cdkn2a*) in satellite cells of geriatric mice causes loss of quiescence and entry into irreversible senescence, demonstrating that the repression of *Cdkn2a* mediated by polycomb proteins is needed to maintain the quiescent state of satellite cells in muscle homeostatic conditions (revised in Sousa-Victor et al., 2015).

Additional methylation events regulate the activity of satellite cells throughout myogenesis. One layer of epigenetic regulation is performed by direct interaction of the arginine methyltransferase Carm1 with Pax7. In quiescent satellite cells Carm1 binding to Pax7 is inhibited; in contrast, when satellite cells are activated, Carm1 interacts and methylates Pax7. Methylated Pax7 directly binds to the Thritorax complex resulting in its recruitment to the Myf5 promoter, leading to H3K4 methylation, Myf5 expression and myogenic commitment (Kawabe et al., 2012). Finally, a very recent study has shown that the histone methyltransferase Suv4-20H1 is necessary to maintain satellite cell quiescence by causing a condensed state of the heterochromatin through the transcriptional repression of MyoD (Boonsanay et al., 2016). Indeed, Suv4-20H1 binds directly to the MyoD Distal Regulatory Region enhancer and catalyzes the transcriptionally repressive H4K20me2 mark to enforce quiescence. Moreover, ablation of Suv4-20H1 specifically in satellite cells resulted in changes in chromatin structure accompanied by increased MyoD expression.

In addition to muscle injury, low stress exercise can also activate satellite cells, via accelerated Wnt signaling (Fujimaki et al., 2014). Indeed, the upregulation of canonical Wnt/β-catenin signaling pathway modifies the structure of chromatin at the *Myod1* and M*yf5* promoters, which results in an increased expression of both genes and a higher number of proliferating satellite cells. Of interest, in a recently published genome-wide analysis of p38α binding at promoters, the Wnt signaling pathway appeared as one of the principal signaling cascades modulated by p38α (Segales et al., 2016). This finding highlights the importance of p38α-dependent regulation of myogenesis, since modulation of Wnt signaling has been shown to critically regulate different aspects of satellite cell functions *in vivo* (expansion, switching from proliferation to differentiation or cell motility; Le Grand et al., 2009; Bentzinger et al., 2014; Murphy et al., 2014).

Interestingly, several recent whole transcriptome analysis of quiescent and proliferating muscle stem cells have shown that the expression of DNA methyltransferases is deregulated, since DNMT1 was markedly increased in activated satellite cells compared to quiescent satellite cells, whereas DNMT3a and all Tet isoforms were downregulated (Pallafacchina et al., 2010; Liu et al., 2013; Ryall et al., 2015a). These observations suggest that a regulation of the DNA methylation status, on top of regulating differentiation (see below) may be critical for initiating the MRF transcriptional program and/or regulating cell cycle in the transition between quiescence and proliferation.

## Epigenetic repression of the myogenic-differentiation gene program in proliferating satellite cells

Epigenetic events are crucial to maintain satellite cells in a quiescent or proliferating state and prevent their premature differentiation (Figure 1). DNA methylation is thought to be one of the main repressive systems acting on muscle gene loci (reviewed in Carrio and Suelves, 2015; Laker and Ryall, 2016). Indeed, DNA methyltransferase inhibitors (such as 5-azacytidine) can induce the transdifferentiation of fibroblasts into myoblasts (Taylor and Jones, 1979; Lassar et al., 1989); moreover, treatment of C2C12 myoblasts with 5-azacytidine promotes myogenesis, resulting in myotubes with enhanced maturity as compared to myotubes from untreated cells (Hupkes et al., 2011). Whereas the DNA methylation status of quiescent satellite cells has not been investigated in high detail, it has been shown that, when myoblasts differentiate into myotubes, a subset of genes display changes in their DNA methylation status, most of them becoming hypomethylated (Carrio et al., 2015). Other studies have proved that DNA methylation helps to restrict myogenin activation until both MEF2A and SIX1 transcription factors are co-expressed, in embryonic myogenesis and in adult myoblasts, and that binding of SIX1 and MEF2A is required for demethylation at the myogenin locus (Palacios et al., 2010b). Additionally, Ezh2 targets the DNA methyltransferases DNMT3a and DNMT3b to specific genes, thus linking the two repressive mechanisms: DNA and histone methylation (Vire et al., 2006). However, it is still unclear how DNA methylation is regulated during myogenesis or if this regulation affects only a subset of genes. A recent study showed the presence of 5-methylcytosine and 5-hydroxymethylcytosine within specific gene regions of the Notch signaling pathway in myoblasts, myotubes and mature skeletal muscle (Terragni et al., 2014). Notch signaling is crucial for the regulation of several developmental stages, including the quiescence and proliferative states of satellite cells (Bjornson et al., 2012; Mourikis et al., 2012; Qin et al., 2013). Terragni et al. identified that several Notch signaling genes, such as *Notch1* and its ligands *Dll1* and *Jag2* were hypomethylated and hydroxymethylated in skeletal muscle lineages, and propose that hypomethylation and/or hydroxymethylation could directly regulate the expression of these genes, thus placing key myogenic processes regulated by Notch signaling (e.g., satellite-cell maintenance and self-renewal; see Mourikis and Tajbakhsh, 2014) under epigenetic control (Terragni et al., 2014). Therefore, is crucial to determine how these modifications are regulated by specific DNA methyltransferases and demethylases that act during myogenesis.

Another important epigenetic mechanism to repress muscle-differentiation gene expression is the post-translational modification of histones. In proliferating satellite cells, the promoters of genes important for muscle differentiation contain histones that are hypoacetylated and marked with H3K9me2, H3K9me3, and H3K27me3. These repression marks are catalyzed by histone deacetylases (HDACs) and histone lysine methyltransferases from the PcG and Suv39H1 families (Palacios and Puri, 2006; Segales et al., 2015). In proliferating myoblasts, the transcriptional regulator Ying Yang 1 (YY1) recruits the histone H3K27 methyltransferase Ezh2 to muscle genes promoters, thus preventing their expression (Caretti et al., 2004). Another lysine methyltransferase, G9a, catalyzes the repressive H3K9me2 mark on MyoD target promoters and also directly methylates MyoD at lysine 104 (K104) to repress its transcriptional activity (Ling et al., 2012).

Muscle-specific gene expression can also be repressed by the action of HDACs, which remove acetyl groups from histone tails and also maintain transcription factors in a deacetylated state. In the absence of promyogenic signals, class I HDACs interact with MyoD and several members of class II HDACs interact with MEF2 repressing its activity, thus avoiding premature expression of skeletal muscle differentiation genes (Lu et al., 2000). Moreover, there is another class of HDACs called Sirtuins (or class III HDACs; Sir2), whose activity depends on NAD^+^ levels and forms a repressor complex with pCAF and MyoD, suggesting that Sir2 works as a redox-sensor regulating muscle-specific genes expression in response to metabolic changes (Fulco et al., 2003). Gene transcription can also be regulated by other chromatin modifications, such as by switching canonical histones with histone variants or expressing specific histone isoforms in a cell type-specific manner (Happel and Doenecke, 2009). For instance, in undifferentiated myoblasts, MyoD expression is repressed by the homeobox protein Msx1, that interacts with the repressive histone methyltransferase G9a and also binds specifically to the histone variant H1b, which is enriched at the core enhancer region of *Myod1* (Lee et al., 2004).

## Epigenetic regulation of skeletal muscle differentiation

For myogenic differentiation to take place, the expression of the differentiation-inhibitory gene program operating during myoblast proliferation has to be repressed. In fact, several cell cycle inactivating mechanisms are known act together with early muscle differentiation events. For instance, cell cycle exit is regulated by the retinoblastoma protein pRb, which represses the E2F transcription factors and its implicated in the recruitment of H3K9me3 and H3K27me3 methyltransferases at cell cycle-associated genes (Blais et al., 2007). Furthermore, the small chromatin-binding protein p8, which interacts with MyoD, the histone acetyltransferase p300 and the RNA helicase p68, negatively regulates the cell cycle and promotes myogenic differentiation (Sambasivan et al., 2009). Additionally, in myoblasts that are induced to differentiate, *Pax7* promoter presents increased levels of H3K27me3 repression mark, mediated by Ezh2 (Palacios et al., 2010a).

Following reception of differentiation-promoting signals, the epigenetic pattern of muscle-specific genes is quickly modified, and the Polycomb-mediated repressive H3K27me3 marks are substituted by the transcriptionally permissive H3K4me3 mark (Figure 1). H3K27me3 removal is mediated by the histone demethylase UTX, which can be recruited to muscle specific loci by the homeodomain transcription factor Six4 and/or the histone chaperone Spt6 (Seenundun et al., 2010; Wang et al., 2013). Importantly, a very recent study using UTX specific deletion in satellite cells demonstrated that demethylation of H3K27 is required for myofiber regeneration (Faralli et al., 2016). Indeed, the demethylase activity of UTX has been proved to be necessary for myogenin expression, which leads to differentiation of myoblasts. Thereby, the authors uncovered a crucial role for the enzymatic activity of UTX in muscle-specific gene activation during tissue regeneration, and a physiological role for active H3K27 demethylation *in vivo* (Faralli et al., 2016). In parallel, the combined action of histone demethylases KDM1A/LSD1A and JMJD2A/KDM4A permits the removal of the H3K9me2/3 repressive marks deposited by KMT1A/Suv39h1 in proliferating myoblasts (Choi et al., 2010; Verrier et al., 2011). Moreover, the expression of several key repressors of transcription, such as the H3K27 methyltransferase Ezh2 and the H3K9 methyltransferase G9a, is downregulated at the onset of muscle differentiation (Asp et al., 2011; Ling et al., 2012). On the other hand, through its association with p38-phosphorylated MEF2D, the TrxG complex is recruited to muscle-specific promoters, such as *myogenin* and *Ckm*, and deposits the transcriptionally permissive mark H3K4me3 (Rampalli et al., 2007; Mckinnell et al., 2008). Other histone methyltransferases are also involved in myogenesis regulation; for instance, expression of the H3K4 histone methyltransferase Set7/9 increases during differentiation, and this is required to express muscle contractile proteins and for myofibril assembly (Tao et al., 2011). In addition, Set7/9 interacts with MyoD to enhance the expression of muscle-specific genes and prevents H3K9 methylation at muscle promoters mediated by Suv39h1. Thus, many mechanisms seem to work coordinately to efficiently remove the transcriptionally-repressive marks.

In undifferentiated myoblasts, muscle gene promoters are partially occupied by inactive transcription factors forming a complex with HDACs and HATs. Active HDACs and Sirtuins deacetylate HATs and inhibit their acetyltransferase activity, as demonstrated for Sirt2 and pCAF/Kat2b (Fulco et al., 2003). Importantly, the redox balance of the myoblast can regulate Sirt2 activity: upon receiving differentiation signals, its [NAD^+^]/[NADH] ratio decreases, leading to Sirt2 inhibition and enabling pCAF to acetylate several target proteins including histones, MyoD and MEF2 (Sartorelli and Caretti, 2005). In a recent study, a role in the regulation of myoblast differentiation has been ascribed to Sirt3, a mitochondrial NAD+ dependent deacetylase (Abdel Khalek et al., 2014). Sirt3 expression increases when C2C12 cells reach confluence and is maintained elevated during differentiation. Indeed, depletion of Sirt3 blocks differentiation, causing high levels of ROS and a decreased manganese superoxide dismutase activity (Abdel Khalek et al., 2014). Notably, sirtuins have also been found to play an important role during muscle stem cell activation, when satellite cells undergo a metabolic switch from fatty acid oxidation to glycolysis. This metabolic reprogramming is associated with reduced intracellular NAD^+^ levels and lower activity of the histone deacetylase Sirt1, which leads to increased H4K16 acetylation and induction of muscle gene transcription (Ryall et al., 2015b). Furthermore, mice with muscle-specific ablation of the Sirt1 deacetylase domain have smaller myofibers and impaired muscle regeneration. Of note, the IGF1/AKT signaling pathway leads to p300 phosphorylation, which promotes its interaction with MyoD and the acetylation of muscle gene promoters (Serra et al., 2007). Thereby, the inhibition of HDACs and Sirtuins is coupled to HAT activation, which results in the activation of muscle transcription factors in response to different extracellular cues. Recent studies have also demonstrated that, upon differentiation, the HAT p300 is recruited at distinct Myod1 regulatory regions, resulting in an increased histone acetylation (Hamed et al., 2013). The authors also show that p300 directly participates in the early regulation of Myod1 enhancer, and shed light on how p300 histone acetyltransferase activity is associated to enhancer activation and, consequently, gene transcription (Hamed et al., 2013).

Chromatin remodeling is also essential for myogenesis, as illustrated by the recruitment of the SWI/SNF chromatin remodeling complex to muscle-specific loci, which depends on p38 MAPK activity (Simone et al., 2004). MyoD and the SWI/SNF subunit BAF60c are found to the promoters of MyoD target genes in myoblasts, previous to activation of transcription, while the ATPase subunit Brg1 is recruited only when cells are induced to differentiate, thus allowing chromatin remodeling and muscle genes transcription (Forcales et al., 2012). Furthermore, the histone arginine methyltransferases Prmt5 and Carm1 are also involved in the recruitment of the SWI/SNF complex to different muscle gene promoters, such as *myogenin* and *Ckm* (Dacwag et al., 2009). Interestingly, it has been recently shown that the regulatory sequences of genes expressed at late time points of myogenesis lie in close physical proximity, despite these genes being located on different chromosomes (Harada et al., 2015). Of note, formation of these inter-chromosomal interactions requires MyoD as well as functional Brg1. The late myogenic gene interactions are associated with the repression of these genes at early times of differentiation, suggesting that this higher-order chromatin organization constitutes a mechanism that contributes to temporal regulation of gene expression during muscle differentiation (Harada et al., 2015).

There is increasing evidence indicating that the substitution of canonical histones with histone variants can also regulate gene expression and muscle differentiation. For instance, the chromatin-remodeling complex SNF2-related CBP activator protein (SRCAP) modulates the replacement of histone H2A for the H2A.Z variant, which is associated with active transcription. At the onset of skeletal muscle differentiation, the SRCAP subunit p18^Hamlet^, which deposites H2A.Z on chromatin, is recruited to the myogenin promoter in a p38 MAPK-dependent manner (Cuadrado et al., 2010). Additionally, upon induction of muscle differentiation, the canonical histone H3 is cleaved and the histone chaperones Asf1 and HIRA recruit the variant histone H3.3 to the *Myod1* promoter, allowing MyoD transcriptional activation. Moreover, histone H3.3 is deposited at myogenin and other muscle-specific promoters thanks to other histone chaperone, Chd2, and MyoD itself (Yang et al., 2011; Harada et al., 2012). Another histone variant that has been proved to be essential for the activation of the myogenic program is the macroH2A1.2 (mH2A1.2), which is characterized by the presence of a ~25 kDa evolutionarily conserved carboxyl-terminal domain called the macro domain. Indeed, activation of muscle enhancers is dependent on mH2A1.2, as it regulates both H3K27 acetylation and recruitment of the transcription factor Pbx1 at prospective enhancers (Dell'orso et al., 2016).

## The p38 MAPK signaling pathway

Cell response to external stimuli requires the integration and activation of intracellular mediators and effectors and signal transduction mechanisms, which strongly depend on post-translational modifications of proteins, among which phosphorylation is particularly relevant. A paradigm of intracellular signaling is the activation of mitogen-activated protein kinases (MAPKs), as they seem to participate in most signal transduction pathways. The MAPK superfamily of intracellular serine/threonine protein kinases is evolutionary conserved, and in mammals it principally includes the extracellular signal regulated kinases (ERKs), the c-Jun N-terminal kinases/stress activated protein kinases (JNKs/SAPKs), the p38 MAPKs, the ERK5 or big MAPKs and atypical MAPKs like ERK3/4, ERK7, and Nemo-like kinase (NLK) (Reviewed in Cargnello and Roux, 2011).

p38, a subgroup of the MAPKs, was firstly described as a transducer of the response to environmental stress conditions and as a critical mediator of inflammatory cytokines, but many different non-stress stimuli can also activate p38 MAPK signaling, leading to the regulation of multiple cellular processes, including senescence, apoptosis, cell-cycle arrest, regulation of RNA splicing, tumor development or differentiation of various cell types such as neurons, adipocytes, cardiomyocytes and myoblasts (reviewed in Cuenda and Rousseau, 2007; Igea and Nebreda, 2015). Mammalian cells have four p38 MAPK family members: MAPK14 (p38α), MAPK11 (p38β), MAPK12 (p38γ), and MAPK13 (p38δ). p38α is ubiquitously expressed usually at high levels, whereas p38β is expressed at lower levels and p38γ and p38δ have more restricted expression patterns (Cuenda and Rousseau, 2007; Cuadrado and Nebreda, 2010). All p38 MAPKs are phosphorylated and activated by the MAPK kinase MKK6. Others p38 MAPK kinases are MKK3, which activates p38α, p38γ, and p38δ, and MKK4 that in some cases can activate p38α. Once activated, p38 MAPKs phosphorylate serine/threonine residues of their substrates, which include several transcription factors as well as protein kinases (see Zarubin and Han, 2005). The identification of physiological substrates for p38α and p38β has been facilitated by the availability of specific pyridinyl imidazole inhibitors, such as SB203580/SB202190 and the reported inhibitor of the four p38 isoforms (BIRB0796) (Kuma et al., 2005; Bain et al., 2007). More than 100 proteins have been found to be directly phosphorylated by p38α and many of them are implicated in the regulation of gene expression (Cuenda and Rousseau, 2007; Cuadrado and Nebreda, 2010). In addition, the p38α pathway can regulate the production of extracellular signaling molecules, such as cytokines, chemokines, and growth factors. Recent reports have provided evidence for a dual role of p38α in regulating cancer progression, as, depending on the cell type and the tumoral stage, it can act either as tumor suppressor or as tumor promoter by facilitating tumor cell survival in response to chemotherapeutic treatments (Reviewed in Igea and Nebreda, 2015).

MAPKs activation has also been related to the differentiation capacity of various stem cell types. Particularly, the p38 pathway acts as one of the main controllers of muscle stem cells' fate decisions (Keren et al., 2006; Lluis et al., 2006; Segales et al., 2015). Indeed, several studies recapitulating myogenesis *in vitro* using different cellular models (myoblast cell lines or satellite cell-derived primary myoblasts) have demonstrated an active role of the p38 MAPK pathway in each myogenic stage, where its function as a regulator of the myoblast proliferation-to-differentiation transition is particularly relevant, as p38 induces cell cycle withdrawal and the expression of muscle-specific genes (Keren et al., 2006; Lluis et al., 2006; Liu et al., 2012; Doles and Olwin, 2015; Segales et al., 2015).

## p38 MAPK signaling in satellite cell-dependent myogenesis

Different stimuli can activate p38 MAPK in satellite cells, including inflammatory cytokines, such as TNF or amphoterin/HMGB1, growth factors such as TGFβ or cell-to-cell contact (reviewed in Guasconi and Puri, 2009; Krauss, 2010). The role of p38 MAPKs pathway during myogenesis has been extensively studied, especially *in vitro*. Pioneer studies from the groups of Cuenda, Bengal and Puri showed the requirement of this pathway for myogenic differentiation *in vitro* through distinct mechanisms (Cuenda and Cohen, 1999; Zetser et al., 1999; Wu et al., 2000). It has been proven that p38α turns off the proliferation-promoting JNK pathway by upregulating the JNK phosphatase MKP-1 leading to downregulation of cyclin D1 expression, cell cycle exit and allowing the onset of muscle differentiation (Perdiguero et al., 2007b). Besides this, independent studies have shown that the p38 MAPK signaling pathway is a critical regulator of skeletal muscle differentiation and fusion. In fact, the fusion of myoblasts into myotubes and the induction of muscle-specific genes are prevented by treatment with the p38α/β inhibitors, whereas forced activation of p38 MAPK by ectopic expression of a constitutively active mutant of MKK6 is sufficient to override the inhibitory factors present in proliferating cells and to induce both the expression of differentiation markers and the appearance of multinucleated myotubes (Lluis et al., 2006). Notably, *in vitro* studies using satellite cells lacking individual p38 family members showed that the four p38 isoforms are not completely redundant during myogenesis, and uncovered a predominant role of the p38α isoform in myogenic differentiation and fusion (Ruiz-Bonilla et al., 2008; Wang et al., 2008; Liu et al., 2012), with p38α regulating the whole myogenic gene expression program at multiple steps (see below) and promoting myoblast fusion by upregulation of tetraspanin CD53 (Liu et al., 2012), with p38γ signaling contributing to proliferation by preventing premature differentiation through induction of a repressive MyoD transcriptional complex (Gillespie et al., 2009), whereas p38β and p38δ seemed to be dispensable for these processes (Perdiguero et al., 2007a; Ruiz-Bonilla et al., 2008).

Importantly, p38α/p38β are also required for activation of quiescent satellite cells and MyoD induction (Jones et al., 2005). Moreover, when satellite cells are activated, signaling by p38α leads to inactivation of tristetrapolin (TTP) and stabilization of MyoD RNA (Hausburg et al., 2015). Activated satellite cells enter the cell cycle and a subset undergoes asymmetric division to replenish the muscle stem cells pool. Interestingly, it has been recently published that p38α/β MAPK are asymmetrically activated in only one daughter cell, in which MyoD is induced, allowing cell cycle entry and generating a proliferating myoblast. In contrast, MyoD induction is prevented in the other daughter cell by the absence of p38α/β MAPK signaling, renewing the quiescent satellite cell pool (Troy et al., 2012).

A role of p38α in skeletal muscle growth and regeneration *in vivo* has also been reported. Using conditional satellite cell p38α-null mice the authors confirmed previous *in vitro* studies and demonstrated that p38α restrains postnatal proliferation and promotes timely myoblast differentiation (Brien et al., 2013). p38α ablation caused a postnatal growth defect together with an augmented number of satellite cells, due to increased progenitor proliferation postnatally. Moreover, muscle regeneration after a cardiotoxin-induced injury was delayed in the absence of p38α, with further enhancement of the satellite cell population (Brien et al., 2013). The absence of p38α was accompanied by increased p38γ phosphorylation, and p38γ inhibition *ex vivo* significantly diminished the myogenic defect. As muscle regeneration *in vivo* can occur quite effectively in the absence of p38γ (Ruiz-Bonilla et al., 2008), but is defective in the absence of p38α (Brien et al., 2013), p38α arises as the master kinase for reprogramming gene expression during proliferation-to-differentiation transition of satellite cells, both *in vitro* and *in vivo*.

Similar to mice, an essential role for p38 MAPK has been demonstrated in the regulation of human satellite cells (huSCs) regenerative capacity (Charville et al., 2015). The authors found that p38 was upregulated in activated muscle stem cells compared with quiescent satellite cells. Moreover, reversible inhibition of p38 in cultured human satellite cells prevented differentiation and promoted expansion of huSCs. These expanded satellite cells showed an enhanced self-renewal and engraftment potential in comparison to freshly isolated satellite cells or cells cultured without p38 inhibitors.

## p38 MAPK signaling in the transcriptional and epigenetic regulation of myogenesis

**Figure 2:**
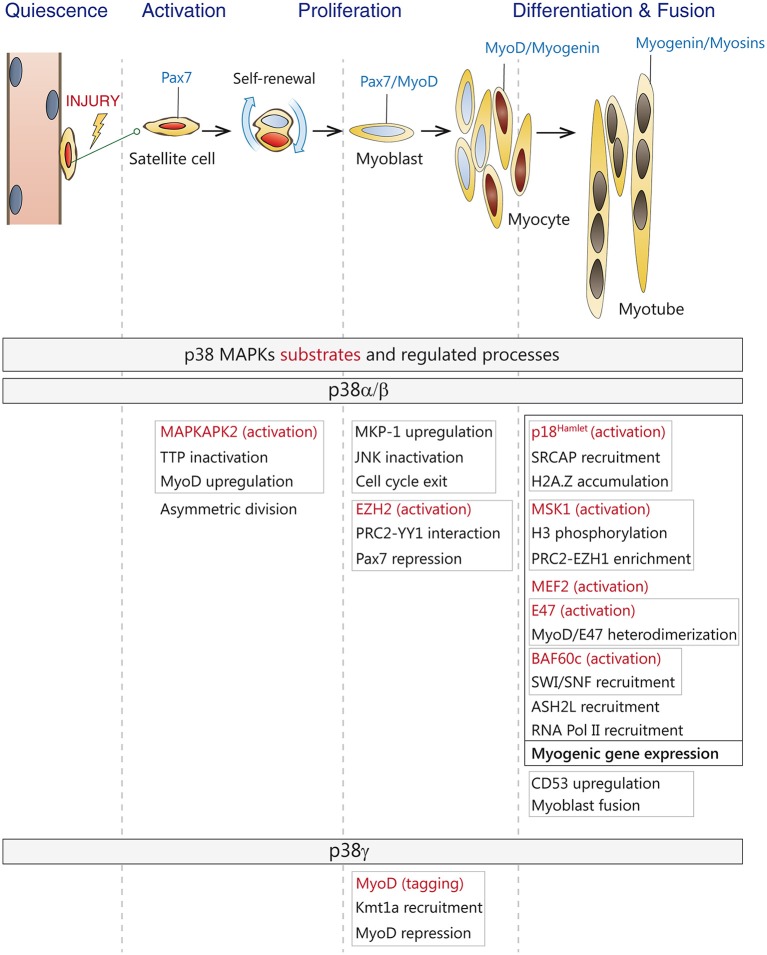
**Roles of p38 MAPKs in satellite cell-driven myogenesis. (Top)** Satellite cells in adult muscles are in a quiescent state. Upon injury, satellite cells are activated, undergo asymmetric division and generate a self-renewing daughter cell and a committed progenitor or myoblast which enters the cell cycle. Myoblasts proliferate, differentiate and fuse to form myotubes and new myofibers during adult muscle regeneration. Canonical markers for each stage are indicated-**(Bottom)** Known substrates and regulated processes by p38α/β and p38γ MAPKs during the different stages of myogenesis. Direct substrates are indicated in red. Linked processes are boxed in gray or black.

p38 MAPK pathway is crucial for the onset of muscle differentiation, as it modulates the expression and/or activity of many of the players involved in the transcriptional and epigenetic regulation of myogenesis (Figure 2). p38α/β MAPKs promote MEF2 transcriptional activity and MyoD/E47 heterodimer formation by direct phosphorylation of MEF2 and E47 (Zetser et al., 1999; Lluis et al., 2005), which in turn enhances RNA Pol II recruitment to myogenic loci, thus initiating the differentiation program. Furthermore, by phosphorylating the chromatin-associated protein BAF60c, p38α/β kinases contribute to the assembly of the myogenic transcriptosome on the chromatin of muscle loci by promoting the recruitment of SWI/SNF chromatin remodeling complex (Simone et al., 2004; Serra et al., 2007; Forcales et al., 2012), and ASH2L-containing mixed-lineage leukemia (MLL) methyltransferase complex (Rampalli et al., 2007). Through phosphorylation, p38α also recruits SNF2-related CBP activator protein (SRCAP) subunit p18^Hamlet^ to muscle loci, which is in turn required for H2A.Z accumulation and transcriptional activation (Cuadrado et al., 2010).

p38α can also repress the expression of Pax7 in differentiating muscle stem cells by phosphorylation of Ezh2, which promotes the interaction between YY1 and PRC2 creating repressive chromatin on the *Pax7* promoter, therefore regulating the decision of satellite cells to proliferate or differentiate (Palacios et al., 2010a; Mozzetta et al., 2011). By contrast, activation of p38γ in satellite cells suppresses MyoD transcriptional activity by direct phosphorylation, via association with the H3K9 methyltransferase KMT1A, thus also affecting this myogenic decision (Gillespie et al., 2009). Moreover, two recent studies have shown that that activation of p38α/β downstream kinase Msk1, via phosphorylation of serine 28 on histone H3, regulates a chromatin exchange between Ezh2- and Ezh1-containing PRC2 complexes at the onset of differentiation (Stojic et al., 2011; Mousavi et al., 2012). Interestingly, downregulation of Ezh1blocks MyoD recruitment to the *myogenin* promoter, thereby impairing muscle differentiation. Furthermore, in differentiating myoblasts, Ezh1-containing complexes have also been associated to Pol II recruitment and transcriptional activation, questioning the prevalent view of PRC2 complexes as chromatin repressors (Mousavi et al., 2012). Thus, depending on the engagement of specific p38 isoforms, the p38 MAPK pathway can either induce or repress gene expression in satellite cells, Interestingly, in addition to all these nuclear functions, p38α has been recently shown to have an unexpected set of cytoplasmic substrates during myoblast differentiation, probably implicated in its own activation (Knight et al., 2012).

In response to osmotic stress, Hog1, the p38 MAPK homolog in yeast, has been shown to activate transcription by acting directly at chromatin (De Nadal et al., 2004; Pokholok et al., 2006). Equally, upon exposure to different kind of stresses, mammalian p38 MAPK can bind to some stress-responsive loci, highlighting that the interaction of the MAPK with target promoters can induce gene expression (Ferreiro et al., 2010). During myogenic differentiation, p38α has also been found to bind certain muscle-specific genes such as myogenin, muscle creatine kinase, and myosin heavy chain (Simone et al., 2004; Palacios et al., 2010a). Importantly, it has been recently demonstrated that p38α exerts its promyogenic function at least in part by binding and acting at chromatin (Segales et al., 2016). Genome-wide localization analysis linked to gene expression profiling have shown that p38α binds to a large number of active promoters during the myoblast proliferation-to-differentiation transition, confirming the importance of kinase signaling pathways in directly regulating transcription (Segales et al., 2016), in agreement with previous reports for other related kinases (Bungard et al., 2010; Tiwari et al., 2012; Di Vona et al., 2015). Interestingly, p38α was also associated to promoters that were transcriptionally inactive or repressed at the onset of differentiation (Segales et al., 2016). Thus, p38α is recruited to a large set of myogenic gene promoters to facilitate their activation or repression, hence pointing to more complex regulatory mechanisms than previously anticipated. How is p38α recruited to muscle loci is not known, but it likely involves interaction with chromatin-regulatory and/or transcription factors, as demonstrated for several stress-induced genes (Ferreiro et al., 2010). Of interest, p38α-bound promoters are enriched with binding motifs for several transcription factors, principally Sp1, Tcf3/E47, Lef1, FoxO4, MyoD, and NFATc, which are known to be phosphorylation substrates of p38 MAPK (Segales et al., 2016).

## p38 MAPK regulation of muscle stem cell functions during aging

Aging is associated with an alteration of organism homeostasis and a progressive decline of tissue functions (Oh et al., 2014). Particularly, in skeletal muscle, mass and strength decline with aging (a process called sarcopenia), which is also linked to a progressive loss of muscle stem cells regenerative capacity. Age-related dysfunction of muscle regeneration has been attributed to both extrinsic alterations in the regenerative microenvironment and to satellite cell-intrinsic mechanisms (recently reviewed in Garcia-Prat et al., 2013; Blau et al., 2015; Brack and Munoz-Canoves, 2015; Sacco and Puri, 2015; Sousa-Victor et al., 2015). Indeed, whereas parabiosis (a process whereby the circulatory system of two living organisms is surgically joined), and whole muscle grafting experiments in mice proved that alterations in the regenerative environment of aged muscles regulate stem cell function (Conboy et al., 2005; Brack et al., 2007), data from recent studies using transplantation of purified satellite cells support cell-intrinsic deficits with aging (Chakkalakal et al., 2012; Bernet et al., 2014; Cosgrove et al., 2014; Price et al., 2014; Tierney et al., 2014; Sousa-Victor et al., 2014a,b; Garcia-Prat et al., 2016). These cell-intrinsic functional deficits can include DNA and oxidative damage, impaired mitochondrial function and alterations of the epigenetic profile, such as post-translational histone modifications or changes in the DNA methylation patterns. In this regard, a progressive increase in DNA methylation in aging muscle has been recently reported (Ong and Holbrook, 2014; Zykovich et al., 2014) and it has also been described that muscle stem cells isolated from aged mice show alterations both in the levels and distribution of the H3K27me3 chromatin mark (Liu et al., 2013). At the level of single genes, the promoter of *INK4a*, which encodes the cell cycle inhibitor and marker of senescence p16^INK4a^, is epigenetically repressed in young quiescent satellite cells via ubiquitination of histone H2A; a mechanism deregulated in geriatric cells, which highly express p16^INK4a^ and undergo a transition to a presenescent state. This conversion from quiescence to senescence is a hallmark of geriatric satellite cells, and it can be reversed through the downregulation of p16^INK4a^ expression, which also restores the self-renewal capacity of satellite cells (Sousa-Victor et al., 2014a,b). In human geriatric satellite cells p16^INK4a^ is upregulated, so these findings are particularly relevant for stem-cell rejuvenation in sarcopenic muscles.

Contrary to other types of stem cells, such as hematopoietic stem cells, both the function and the number of satellite cells decline with aging, compromising the recovery capacity of sarcopenic muscles in response to injury (Brack et al., 2005; Chakkalakal et al., 2012; Sousa-Victor et al., 2014b). Whether satellite cell decline might contribute to age-associated loss of muscle mass in the absence of muscle damage is a matter of active debate. By depleting satellite cells in young sedentary animals, one study showed that these cells do not contribute to the maintenance of muscle size or fiber type composition during aging, but that their loss may contribute to age-related muscle fibrosis (Fry et al., 2015). Through genetic lineage experiments, another study showed that even in the absence of injury, satellite cells contribute to myofibers in all adult muscles, although the extent and timing differed (Keefe et al., 2015). However, genetic ablation experiments in this latter study showed that satellite cells are not globally required to maintain myofiber size of uninjured adult muscle.

Several signaling pathways have been found altered in satellite cells from aged mice, among them fibroblast growth factor receptor-1 (FGFR1), p38 MAPK, and JAK/STAT, thus contributing to impaired control of quiescence and compromised self-renewal capacity (Bernet et al., 2014; Cosgrove et al., 2014; Price et al., 2014; Tierney et al., 2014).

There is clear evidence that during aging the FGFR1 signaling pathway is altered. It has been suggested that increased FGF2 signaling in aged muscle can cause the disruption of satellite cell quiescence (Chakkalakal et al., 2012). In more recent studies it has been proposed that the augmented FGF2 in the aged satellite cell niche constitutes a mechanism to compensate for the loss of FGFR1 signaling. Furthermore, alterations in FGF2 signaling, together with elevated levels of TNF observed in old muscles, have been associated to constitutive and aberrant activation of the p38 MAPK pathway, which leads to impaired self-renewal of aged muscle stem cells (Bernet et al., 2014). Indeed, it has been proved by two different groups that this aberrant activation of the p38 MAPK in aged satellite cells interferes with asymmetric division, resulting in highly reduced self-renewal and regenerative capabilities. Interestingly, pharmacological inhibition of p38α/β can partially restore the proliferative capacity and self-renewal of aged muscle stem cells as assessed in muscle transplantation experiments (Bernet et al., 2014; Cosgrove et al., 2014). Of note, the advantageous effects of p38α/β MAPKs neutralization were strongly enhanced if satellite cells treated with p38 MAPK chemical inhibitors were cultured in a hydrogel matrix, mimicking the biomechanical properties of young muscles, thus providing the basis for improving stem cell engraftment for muscle regeneration in aged individuals (Cosgrove et al., 2014).

Finally, the reduced regenerative potential of aged muscle stem cells has also been correlated with increased JAK/STAT signaling, which impairs satellite cell function by stimulating asymmetric division (Price et al., 2014). Moreover, IL-6-activated STAT3 regulates the expression of the myogenic factor MyoD, promoting differentiation of satellite cells in detriment to their expansion (Tierney et al., 2014). Thus, these studies evidence that intracellular signaling pathways, such as JAK-STAT and p38 MAPK might play distinct age-dependent roles in myogenesis.

## Perspective

As discussed in this review, an emerging concept in the field of myogenesis is that the cell-intrinsic signaling networks that are activated in response to external cues are dysregulated in satellite cells with aging. One paradigm of this age-dependent dysregulation is the p38 MAPK signaling pathway. While it is transiently required for the activation, cessation of proliferation and initiation of the myogenic-differentiation gene program of adult satellite cells, thus playing an important positive role for muscle regeneration in the adult, its persistent activation in aged satellite cells has deleterious consequences on the functionality of these cells, thus impairing efficient regeneration with aging. Thus, identifying the epigenetic and transcription drivers and targets of the p38 MAPK pathway (and of other major myogenic signaling cascades) will be key to shed light on the complex regulation of myogenesis throughout life, and will facilitate the identification of molecular targets that can be druggable for intervening in muscle aging and disease, in order to enhance regeneration of degenerating muscle in both conditions.

## Author contributions

JS, EP, and PM wrote the manuscript. All authors read and approved the final manuscript.

### Conflict of interest statement

The authors declare that the research was conducted in the absence of any commercial or financial relationships that could be construed as a potential conflict of interest.
